# Model of psychosocial determinants of health in processes of social exclusion

**DOI:** 10.3389/fpubh.2023.1156569

**Published:** 2023-06-26

**Authors:** Jessica Cantos-Egea, Jordi Tous-Pallarés, Juana-María Tierno-García

**Affiliations:** ^1^Departament de Pedagogia, Universitat Rovira i Virgili, Tarragona, Spain; ^2^Departament de Psicologia, Universitat Rovira i Virgili, Tarragona, Spain

**Keywords:** social exclusion, health inequity, psychological well-being, health, psychosocial determinants

## Abstract

The substantial increase in the number of families facing social exclusion in Europe and its direct relationship with health inequities is a challenge for studies approaching the social determinants of health and policies dealing with welfare and social inclusion. We start from the premise that reducing inequality (SDG10), has a value and contributes on other goals such as improving health and well-being (SDG3), ensuring quality education (SDG4), promoting gender equality (SDG5) and decent work (SDG8). In this study, we identify disruptive risk factors and psychological and social well-being factors that influence self-perceived health in trajectories of social exclusion. The research materials used a checklist of exclusion patterns, life cycles and disruptive risk factors, Goldberg’s General Health Questionnaire (GHQ-12), Ryff’s Psychological Well-being (PWB) Scale and Keyes’ Social Well-being Scale. The sample consists of 210 people (aged between 16 and 64  years): 107 people in a situation of social inclusion and 103 people in a situation of social exclusion. The data treatment involved statistical analysis, including correlation study and multiple regression analysis, aimed at developing a model of psychosocial factors that may act as health modulators, considering social factors as predictors in the regression model. The results showed that individuals in the sample, in a situation of social exclusion, have a greater accumulation of disruptive risk factors, and these are related to having fewer psychosocial and cognitive resources to cope with stressful situations: less self-acceptance, less mastery of the environment, less purpose in life, less level of social integration and social acceptance. Finally, analysis showed that in the absence of social integration and purpose in life, self-perceived health statuses decline. This work allows us to use the model obtained as a basis for confirming that there are dimensions of psychological and social well-being that should be considered stress-buffering factors in trajectories of social exclusion. These findings can help design psychoeducational programs for prevention and intervention with the aim of improving psychological adjustment and health states, as well as to promote proactive and reactive policies to reduce health inequalities.

## 1. Introduction

Current macroeconomic factors have created new scenarios of inequality and have led many families to situations of social exclusion. Spain has not been left out of these trends and is even above the European average and can therefore be considered one of the most unequal countries in the EU.

This is indicated by multiple data from which we highlight two of those provided by the INE (National Statistics Institute): Spain closed 2021 with 21.7% of its inhabitants at risk of poverty, i.e., people living in households whose income is less than 60% of the national income (Poverty Threshold) and a poverty and social exclusion rate of 27.8%. Regarding inequality, the S80/S20 coefficient (2020 data) indicates that the average income obtained by 20% of the population with the highest the average income obtained by 20% of the population with the lowest income. These and other indicators point to a weakness in our social protection system and are compelling reasons to emphasize the need to address poverty and social exclusion as part of the Sustainable Development Goals in the 2030 Agenda.

In the second half of the 20th century, with the Welfare State in the background, a new epidemiological approach to the Social Determinants of Health (SDH) emerged in line with the postulates of Sigerist (1) and Dunn (2) on the positive nature of health. The first published report that referred to the Social Determinants of Health (SDH) was The Black Report in 1980. It was the first time that a Western government had explicitly exposed the evolution of social inequalities of health in its population. Its findings developed a widespread conviction across Europe of the importance of studying and reporting on social inequalities in different countries.

Over the past 30 years, many authors have recognized the relationship between social determinants of health and how they influence people’s daily living conditions, well-being, and health states (3–9). The SDH approach recognizes the role of social inequalities in health and advocates that health states are related to the opportunities and resources that people have according to their social class, gender, territory, or ethnicity (10, 11).

Other authors argue that inequalities occur when the state of health among individuals and populations are inevitable consequences of genetic differences, social and economic conditions, or lifestyle choices. In contrast, these same authors add that, when discussing the concept of inequity, it is closely linked to access to opportunities to maximize health states. It should not be influenced by social position or other socially determined circumstances (8, 12).

Zuckerman, Oliver, Hollingsworth and Austrin (13) stated that those who experienced negative events were at greater risk of manifesting mental health problems and illnesses. On the other hand, other studies recognize that when people are not able to control the resources needed to cope with adversity, the degree of well-being declined (14, 15).

A model related the social structure to different levels of mediation that influence mental health states was proposed by Barrón and Sánchez (16). According to this model, based on the socio-economic and socio-structural position of the person, situations of alienation are produced, which would correspond to the set of social support relationships that enrich the maintenance of the health of individuals and facilitate adaptive behaviors in stressful situations. On the other hand, environmental factors converge with the set of risk factors that can generate stressful situations. On a second level, psychological and psychosocial mediations are recognized through which the psychological effects of stress are modulated: coping styles, self-esteem and social support, which play an important role as modulators in the states of psychological well-being, mental health and in the processes of depression.

**Figure 1:**
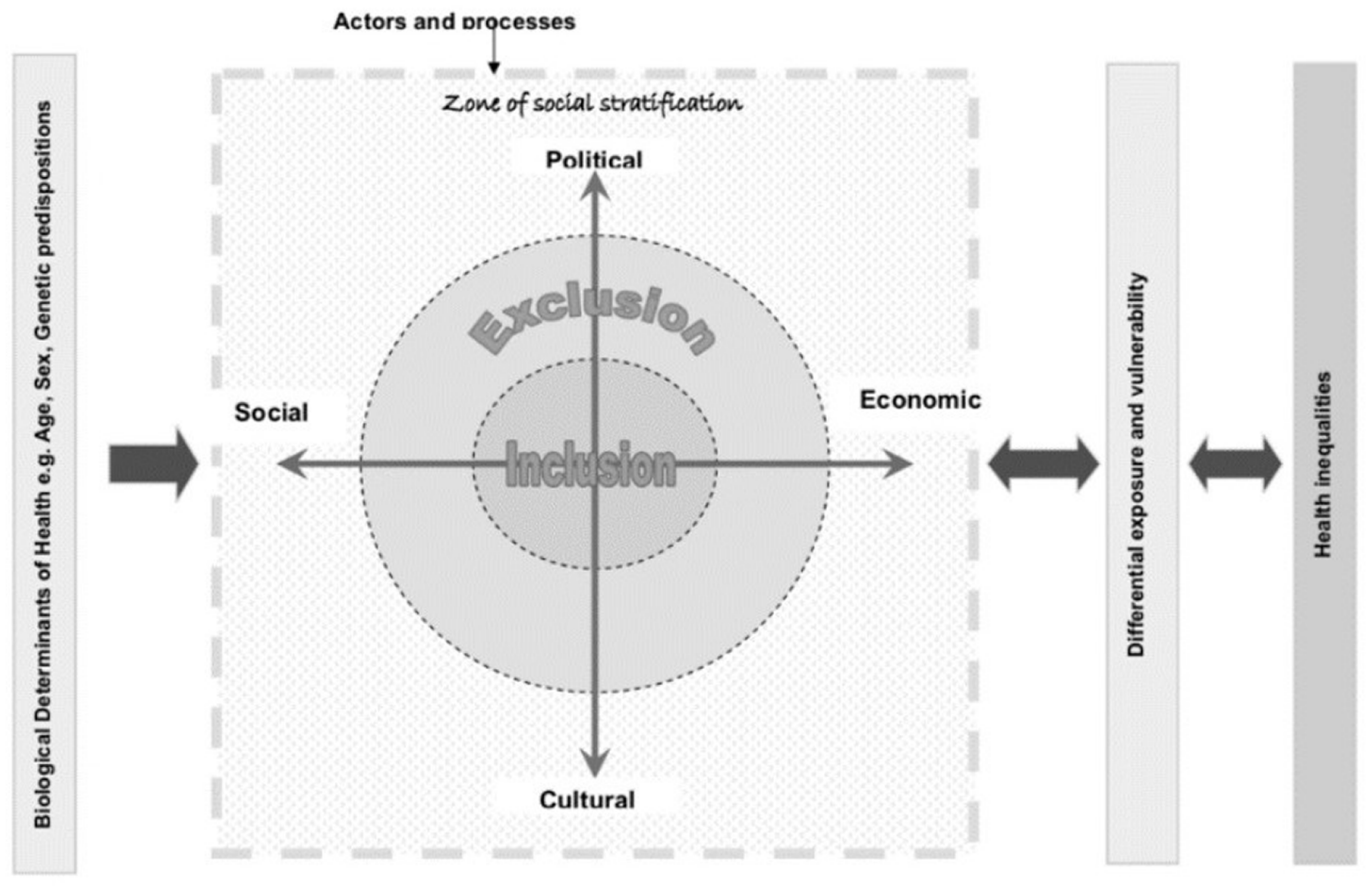
The social exclusion knowledge network [WHO (1): p. 38].

The Commission on Social Determinants of Health developed the Social Exclusion Knowledge Network (SEKN) model (Figure 1), which sheds light on the correlation between social inclusion/exclusion and health inequalities (17). The model delves into the unequal power relations that exist in four social dimensions, namely political, social, cultural and economic, and highlights the dynamics that result in differential exposure to health status.

As per the SEKN model, the differences in power exercised by various dimensions, along with the influence and opportunities they provide, result in hierarchical systems of social stratification based on gender, ethnicity, class, caste and age, referred to as biological determinants of health. These systems of stratification and unequal access to power and resources result in differential exposure to health-damaging circumstances, which reduces people’s capacity to protect themselves from such circumstances and restrict their access to essential health and other services. This in turn generates health inequalities, which feed back into the system, thereby further increasing inequalities and making people more vulnerable and exposed (17).

The model highlights that the impact of social exclusion processes on health inequalities is both constitutive and instrumental. Simple restriction of participation in social rights or lack of equal opportunities and resources in different dimensions (economic, social, political and cultural) leads to greater negative impacts on health and well-being (17).

Robitschek and Keyes (18) stress the importance of well-being as a protective factor against mental disorders and argue that individuals with high levels of psychological and social well-being have a higher quality of life and improved mental health.

Dodge et al. (19) posit that well-being is determined by the correlation of life challenges and available resources. Based on Headey & Wearing (20), dynamic equilibrium theory of well-being, Hendry & Kloep (21), developmental life model, and Cummins’ (22) theory, they define well-being as a state of equilibrium affected by life events and challenges and the set of resources available to the individual. When individuals have more resources than challenges, they experience stable well-being, but when they have more challenges than resources, their well-being becomes unbalanced.

Ryff and Singer (23) understand well-being as an element that values a person’s positive state of mind, personal growth, and the ability to meet life’s challenges. Lyubomirsky et al. (24) recognize that individuals with high positive health states have better psychological functioning, longer life expectancy, better physical health, and high-quality interpersonal relationships. Blanco and Díaz (25) and Bilbao (26) associate psychological and social well-being with positive mental health and its dimensions as indicators of good psychological and social adjustment.

Therefore, positive states of mind are linked not only to a fuller life but also to a healthier existence, which favors social inclusion (24). This study aims to examine the relationship between psychological and social well-being and social exclusion, identify psychosocial and cognitive resources that hinder optimal psychological functioning in life trajectory processes in social exclusion, and create psycho-educational programs that reinforce healthy buffer factors in health, with a focus on vulnerable groups and reducing health inequalities. The study aims to contribute to achieving the Sustainable Development Goals (SDGs), because we start from the premise that reducing inequality (SDG10), has a value and contributes on other goals such as improving health and well-being (SDG3), ensuring quality education (SDG4), promoting gender equality (SDG5) and decent work (SDG8).

## 2. Materials and methods

### 2.1. Participants

The study examines a sample of 210 individuals, between the ages of 16 and 64 years. The sample consisted of two groups: 107 individuals who were in a situation of social inclusion, and served as the control group (21.5% men and 78.5% women) and 103 persons in a situation of social exclusion (35% men and 65% women), with an average age of 43 and, regarding their origin, 62.1% of the participants were native and 37.9% were foreign-born.

### 2.2. Instruments

To assess the variables under study (disruptive risk factors, psychological well-being, social well-being, and self-perceived health), four assessment instruments were administered: 1 checklist, 1 questionnaire and 2 scales.

Checklist of exclusion patterns, life cycles and disruptive risk factors (CDRF) [Diputació (27)]: It contains 60 disruptive risk factors to identify moments of rupture that can affect people in different phases of the life cycle.

The Goldberg’s General Health Questionnaire (GHQ-12) (28) is a self-administered screening tool used to measure the psychological well-being and identify the presence of psychological distress in individuals. The GHQ-12 consists of 12 items that measure four domains of distress, including depression, anxiety, social dysfunction, and loss of confidence. Responses are scored on a four-point Likert scale, and the total score ranges from 0 to 36, with higher scores (> 14) indicating greater psychological distress. The GHQ-12 has demonstrated good internal consistency, with Cronbach’s alpha coefficients ranging from 0.78 to 0.95 across studies. It has also been found to have good sensitivity and specificity in detecting mental health problems, with cutoff scores ranging from 9 to 12. Furthermore, the GHQ-12 has been validated in different cultural contexts, indicating its cross-cultural validity.

Ryff’s Psychological Well-being Scale (PWS) (29): It is a scale that is used to measure psychological well-being through six subscales with twenty-nine items. The response format has scores ranging from one (strongly disagree) to six (strongly agree). The variables they measure are: Self-acceptance (SF), Positive relationships with others (PR), Autonomy (AU), Environmental mastery (EM), Purpose in life (PL) and Personal growth (PG). Subscales show acceptable internal consistency (with values between 0.71 and 0.83), except for Personal growth, which has a lower consistency (*α* = 0.68), so it has not been considered in the present study.

Keyes’ Social Well-being Scale (SWS) (25): It is a scale used to assess the perception of the five aspects of the social environment that facilitate psychological well-being: Social integration, Social acceptance, Social contribution, Social actualization and Social coherence. In this study, only the Social integration (SI), Social acceptance (SA) and Social contribution (SC) factors have been used. Two of them subscales have acceptable values of internal consistency: Social acceptance (*α* = 0.79) and Social contribution (*α* = 0.80). Given that in previous studies (25), the Social integration subscale showed a poor internal consistency (*α* = 0.67), we proceeded to calculate the internal consistency with our samples, which was somewhat higher (*α* = 0.71). This value indicates a low correlation between item and total, but acceptable according to the established criteria.

### 2.3. Procedure

In this study, purposive sampling was used to select the sample of individuals in a situation of social exclusion. This sampling technique involves the intentional selection of participants based on specific criteria, rather than random selection. In this case, experts selected participants based on defined exclusion and inclusion criteria.

**Figure 2:**
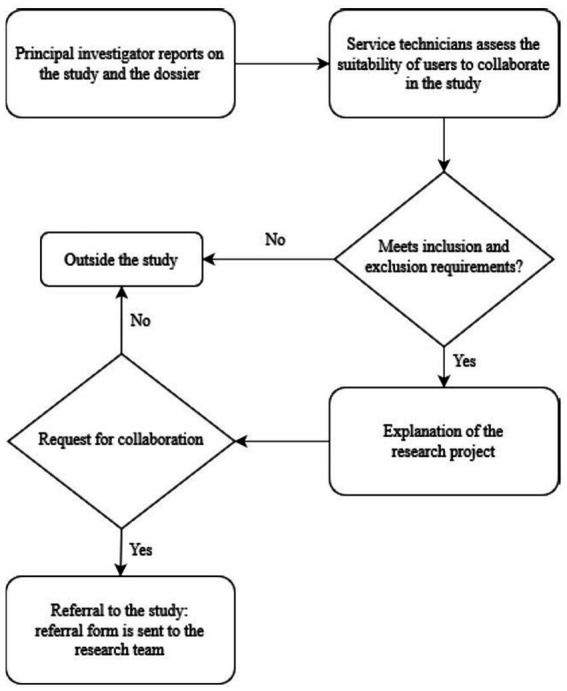
Selection procedure for the social exclusion sample.

Figure 2 below illustrates the selection process of the sample of individuals in a situation of social exclusion. All participants were at risk of social exclusion based on the AROPE criteria, with 98% of them experiencing severe material deprivation (*n* = 101), 84.5% at risk of relative poverty (*n* = 87), and 62% in a situation of very low labor intensity (*n* = 64). All participants were referred by third sector entities (57%) or social services (43%).

On the other hand, the sampling of the social inclusion sample was non-probabilistic by quotas. This means that participants were chosen based on specific criteria to obtain two samples with comparable socio-demographic profiles.

**Figure 3:**
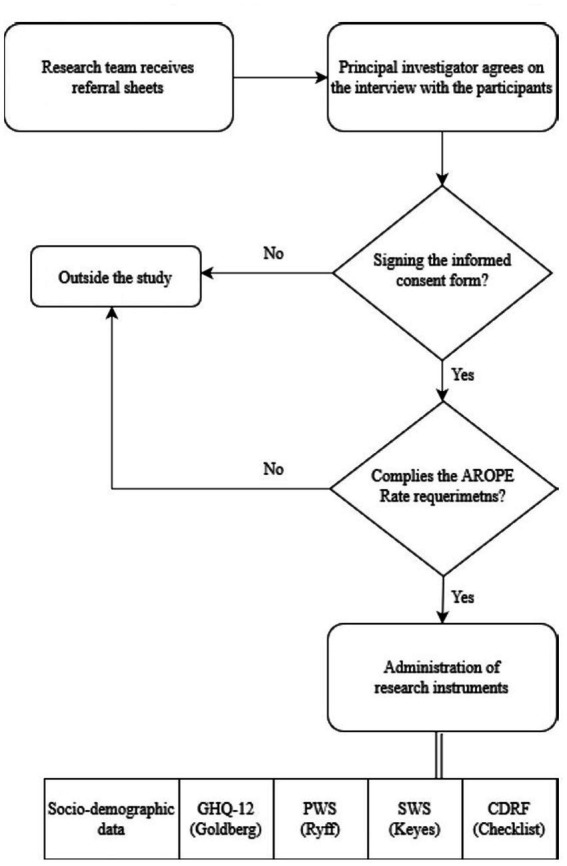
Procedure for the exploratory phase of the social exclusion sample.

After that, using a descriptive and correlational cross-sectional design, in the first phase, an informative meeting was held with the managers and coordinators of the institutions and entities collaborating in the study and they were sent an informative dossier containing all the information and the procedure to be followed to refer the participants. Subsequently, members of the research team went to the different institutions to conduct individual interviews lasting between 60–90 min. All participants signed the letter of informed consent. The study complied with the ethical values required in the framework of this research (Figure 3).

### 2.4. Data analysis

With a mainly quantitative approach, data processing is based on a correlational study. The temporal scope of the research is cross-sectional. Data were analyzed using SPSS 25 statistical software.

Firstly, descriptive statistics of the socio-demographic variables and of data obtained from each instrument used for the study were calculated (minimum, maximum, mean, median, and standard deviation).

On the other hand, the Kolmogorov–Smirnov test was performed to check whether the distribution of the samples was normal or not. The results showed that we were working with non-normal samples and therefore non-parametric tests were chosen for further data analysis. Subsequently, significant differences between the two samples were identified using the Mann–Whitney *U* test and Spearman’s rho and stepwise multiple regression analyses were carried out in order to analyze the following situations:

- Existence or not of significant differences between the inclusion and social exclusion samples in the scores of self-perceived health, psychological well-being, social well-being, social well-being, psychological well-being and social exclusion.
- Relationship between the variables of psychological well-being, social well-being and self-perceived health in the social exclusion sample.
- Predictive models of self-perceived health through the analysis of the factors of psychological well-being and social well-being in socially excluded people.

## 3. Results

The results showed that people in a situation of social exclusion, in contrast to socially included people, have a higher accumulation of disruptive risk factors, levels of self-perceived health or a high level of psychological distress and fewer psychosocial and cognitive resources.

Firstly, a descriptive analysis of the disruptive risk factors that arise in the life cycle of socially excluded people was carried out (mean = 28.3, median = 28, minimum = 11, maximum = 47 and standard deviation = 7.7). The most recurrent disruptive risk factors in trajectories of social exclusion are difficulties in paying household expenses (94.2%), as more than 84% have to constantly resort to social benefits in order to subsist. Other disruptive risk factors are also recurrent:

- At some point been unemployed (89.3%)
- Having been hired under precarious conditions (88.3%)
- Lack of the right training to get a better job (86.4%)
- Lack of a solid relational network and having suffered a significant emotional break (83.5%)
- A rupture with their relational network (81.6%)
- Having been unemployed in the long-term (79.6%)
- Lost the job at least once (79.1%)

A sequential pattern of the most common disruptive risk factors can be deduced: people in a situation of social exclusion have greater difficulty in satisfying basic needs and resort to a chronification of social assistance, are constantly in a situation of unemployment or precarious employment or in unstable and temporary jobs, have a strong lack of specialized training and have a very weak or scarce relational network.

Secondly, the Mann–Whitney *U*-test was used to check whether there were significant differences between the two samples studied in relation to self-perceived health, psychological and social well-being. In this sense, Table 1 identifies the variation in the levels of self-perceived health in M1 and M2, obtaining a value (*U* = 960.50, *p* = 0.000), which implies the existence of significant differences between the two samples (M1 and M2). The results show that self-perceived health is worse in people in a situation of social exclusion than in people in a situation of social inclusion.

**Table 1:** Mann–Whitney *U*-test on self-perceived health in the socially included sample (M1) and in the socially excluded sample (M2).

Sample	*n*	Average range	Range sum	Mann–Whitney’s *U*-test	Asymptotic significance (2-tailed)
Self-perceived health					
M1	107	62.98	6,738.50	960.50	0.000
M2	103	149.67	15,416.5		

Tables 2, 3 show the variation of the different factors of psychological and social well-being, obtaining significant scores in all values: self-acceptance (*U* = 1,981.50, *p* = 0.000), in positive relationships (*U* = 1,568, *p* = 0.000), in autonomy (*U* = 3,100. 50, *p* = 0.000), in mastery of the environment (*U* = 1,365, *p* = 0.000), in personal growth (*U* = 3,323, *p* = 0.000), in purpose in life (*U* = 1,976, *p* = 0.000), in social integration (*U* = 3,419.50, *p* = 0.000), in social acceptance (*U* = 1,038, *p* = 0.000) and in social contribution (*U* = 4,297.50, *p* = 0.006).

**Table 2:** Mann–Whitney’s *U*-test on psychological well-being in the socially included sample (M1) and the socially excluded sample (M2).

Sample	*n*	Average range	Range sum	Mann–Whitney’s *U*-test	Asymptotic significance (2-tailed)
Self-acceptance					
M1	107	138.48	14,817.50	1981.50	0.000
M2	103	71.24	7,337.59		
Positive relationships with others					
M1	107	142.35	15,231	1,568	0.000
M2	103	67.22	6,924		
Autonomy					
M1	107	128.02	13,698.50	3,100.50	0.000
M2	103	82.10	8,456.50		
Environmental mastery					
M1	107	144.24	15,434	1,365	0.000
M2	103	65.25	6,721		
Personal growth					
M1	107	125.94	13,476	3,323	0.000
M2	103	84.26	8,679		
Purpose in life					
M1	107	138.53	14,823	1,976	0.000
M2	103	71.18	7,332		
General psychological well-being					
M1	107	146.22	15,645.50	1,153.50	0.000
M2	103	63.20	6,509.50		

**Table 3:** Mann–Whitney’s *U*-test on social well-being in the socially included sample (M1) and the socially excluded sample (M2).

Sample	*n*	Average range	Range sum	Mann–Whitney’s *U*-test	Asymptotic significance (2-tailed)
Social integration					
M1	107	125.04	13,379.50	3,419.50	0.000
M2	103	85.20	8,775.50		
Social acceptance					
M1	107	147.30	15,761	1,038	0.000
M2	103	62.08	6,394		
Social contribution					
M1	107	116.84	12,501.50	4,297.50	0.006
M2	103	93.72	9,653.50		

There are significant differences in all the variables of the psychological well-being scale (Ryff) and social well-being (Keyes). People in a situation of social inclusion (M1) have higher scores of self-acceptances, have a greater capacity to maintain their independence and personal autonomy, show a greater purpose in life and better management of the environment and the potential to grow as a person. People in a situation of social inclusion have more solid and trusting relationships with their social environment. However, people on trajectories of social exclusion (M2) report lower scores in perceived self-acceptance, autonomy, purpose in life, mastery of the environment and capacities for personal growth. In addition, they lack close personal relationships that could enrich their lives. Socially excluded people (M2) have lower levels of trust, acceptance, and positive attitudes toward people in their immediate environment and report feeling like the less active members of the society or community to which they belong.

As can be seen, socially excluded people are characterized by an accumulation of disruptive risk factors, high levels of psychological distress and low scores on psychological and social well-being. To explore, more specifically the relationship between self-perceived health, psychological and social well-being factors and the accumulation of disruptive risk factors in the socially excluded sample, we have calculated Spearman’s correlation between all these variables.

Thus, Table 4 shows a positive correlation between the score of the global indicator of self-perceived health and the score of the global indicator of accumulation of disruptive factors (r_S_ = 0.234; *p* < 0.05). There are negative correlations between the overall disruptive risk factor accumulation with three factors of the psychological well-being scale: self-acceptance (r_S_ = −0.365; *p* < 0.01), environmental mastery (r_S_ = −0.254; *p* < 0.01), purpose in life (r_S_ = −0.321; *p* < 0.01) and global psychological well-being scores (r_S_ = −0.302; *p* < 0.01) and with two factors of the social well-being scale: social integration (r_S_ = −0.375; *p* < 0.01) and social acceptance (r_S_ = −0.239; *p* < 0.05).

**Table 4:** Correlation (Spearman’s rho) between the accumulation of global disruptive risk factors and self-perceived health, psychological well-being factors, and social well-being factors in people in socially excluded situations.

	Global disruptive risk factors
Self-perceived health (SH)	0.234*
Psychological well-being factors	
Self-acceptance (SF)	−0.365**
Positive relationships with others (PR)	−0.070
Autonomy (AU)	−0.183
Environmental mastery (EM)	−0.254**
Personal growth (PG)	0.003
Purpose in life (PL)	−0.321**
Global psychological well-being	−0.302**
Social well-being factors	
Social integration (SI)	−0.375**
Social acceptance (SA)	−0.329*
Social contribution (SC)	−0.132

**p < 0.01; *p < 0.05. *N* = 103.

Bold values indicates the highest values.

Therefore, it is concluded that socially excluded people have a higher accumulation of disruptive risk factors and these are related to having fewer psychosocial and cognitive resources to cope with stressful situations: less self-acceptance, less environmental mastery, less purpose in life, lower level of social integration and social acceptance.

Finally, a stepwise multiple regression was carried out to establish a predictive to identify which factors of psychological well-being and social well-being are better predictors of self-perceived health states (Table 5).

**Table 5:** Predictive model of the self-perceived health of people in a situation of social exclusion by analyzing factors of psychological well-being and social well-being.

	Self-perceived health	
	Beta	Beta
Social integration (SI)	−0.381^*^	−0.266^*^
ΔR^2^ =	0.136	
*F* (1, 101) =	17.103	
Purpose in life (PL)	–	−0.226^*^
ΔR^2^ =	0.038	
R^2^adj.=	0.167	
*F* (2, 100) =	11.197	

Stepwise multiple regression. ^*^p < 0.001. *N* = 103.

The results of the stepwise regression analysis indicate that, in the social exclusion sample, the combination of two factors (low social integration and low purpose in life) implies a higher risk of suffering psychological distress or low self-perceived health, with an explained variance of 18.3%.

## 4. Discussion

The study aimed to analyze the connection of psychosocial factors (disruptive risk factors, psychological well-being, and social well-being) with self-perceived health. In particular, the research aims to clarify which psychosocial factors influence self-perceived health on the trajectory of social exclusion and being able to recognize buffer factors to reduce social inequalities in health.

Firstly, the high average number of disruptive risk factors in social exclusion trajectories indicates that socially excluded people accumulate disruptive risk factors in different life domains. In fact, the results show that there are axes of inequality and combinations of disruptive risk factors that are more recurrent in the social exclusion sample. These data are in line with the results that showed that the main axes to prevent social exclusion should be oriented toward the promotion of occupation insertion, social relations and integration in the community (30–32). This brings us back to the key concepts of community action, social citizenship and governance.

Results also confirmed that the mean psychological health score in the social exclusion trajectories is higher than in the social inclusion sample, with scores higher than 14 points reflecting a low level of self-perceived health. This difference between the two samples is indeed significant. We can corroborate that this sample manifests a very low self-perceived health; therefore, a high level of psychological distress.

Socially excluded people score lower in all the factors of psychological and social well-being, in the same way as Ryff (33) approach: socially excluded people have a lower perception of self-acceptance, less autonomy, less purpose in life, less mastery of their environment and less capacity to develop personal growth. In addition, they do not have close personal relationships that enrich their lives, nor do they feel socially accepted. Similarly, the results obtained show that socially excluded people have less confidence, acceptance and positive attitude toward others and report feeling not very active members of the society or community to which they belong.

No studies have been found that explicitly establish a concrete relationship between the different factors of psychological well-being and the processes of social exclusion as we propose in this study. Even so, in the socio-psychological model of Barrón and Sánchez (16) it is recognized that, just as the social position (influenced by social and environmental factors) influences mental health and depressive states, it also influences psychological well-being. Evidence has been found that people who are socially excluded score low levels of psychological well-being (34). In this way, it is concluded that people in social exclusion trajectories mobilize fewer psychosocial and cognitive resources compared to those in a situation of social inclusion is ratified. As a result, they may have difficulties in maintaining an optimal level of psychological and social well-being.

Secondly, the results have confirmed that the higher the accumulation of disruptive risk factors, the lower the levels of psychological and social well-being and the poorer the self-perceived health. Therefore, it is found that the accumulation of disruptive risk factors is related to levels of self-perceived health. On the other hand, in relation to the psychological and social well-being variables, the accumulation of disruptive risk factors is generally related to the manifestation of less self-acceptance, less mastery of the environment, less purpose in life and lower levels of integration. These results are congruent with those found by Lupien et al. (35) when they argued that the greater the accumulation of life events, the more changes, or readjustments to the stressful situation, and, thus, the greater the impact 0 on psychological adaptive processes and well-being. Along the same lines (36), and Peterson (37) show how residents of disadvantaged areas have lower levels of integration and greater stressful experiences.

Having determined the relationship between self-perceived health scores and psychological and social well-being scores, the last step was to explore whether there were psychological and social well-being factors, as well as axes of inequality, that explain greater variance between self-perceived health in the trajectories of social exclusion and social inclusion.

In the trajectories of social exclusion, it is shown that states of psychological distress are worse when a person is not socially integrated, has no purpose in life and does not have social networks to support them and a sense of social inclusion, states of psychological distress are worse. These findings are consistent with Frankl (38), who stresses that having a strong purpose in life is a very important dimension of human existence to provide people with a sense of vitality, motivation and resilience. Furthermore, Barrón and Sánchez (16) define integration as a positive determinant of health states.

**Figure 4:**
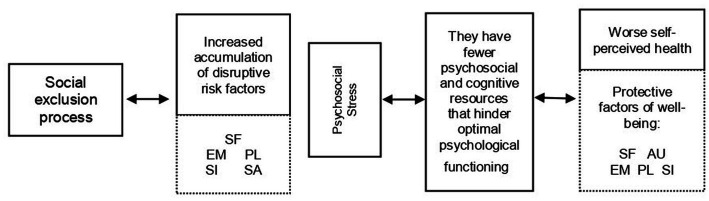
Model of psychosocial determinants of health in processes of social exclusion.

With all these results, a model of psychosocial determinants of health in processes of social exclusion is proposed, highlighting the protective factors of health that can become resilient resources (Figure 4).

Firstly, the set of disruptive risk factors that arise in the life cycle: people in a situation of social exclusion face a greater number of disruptive risk factors, which require different types of response, so they have to mobilize their resources to act effectively in the face of psychosocial stress.

Secondly, it is shown that accumulating more risk factors is related to having fewer psychosocial and cognitive resources that hinder good psychological functioning: poor self-acceptance, poor perceived mastery of the environment, lack of direction with life purpose, few or no positive social relationships, and a feeling of non-integration and social acceptance.

The model ends by exposing those factors that should be considered as protective of well-being (self-acceptance, autonomy, mastery of the environment, purpose in life and social integration) and highlights those healthy coping styles (focus on the solution of the problem, positive reappraisal and search for social support), which would favor the improvement of health states, and those that have turned out to be maladaptive (avoidance).

Based on the above, there are elements to affirm that there are psychosocial factors that play a determining role in health states both in trajectories of social inclusion and exclusion. The results of this study modestly add to the incipient line of work that claims the salutogenic approach based on Positive Psychology. This epistemological framework emphasizes the positive aspects of people and those factors that favor adaptation to adverse life events and improve quality of life and well-being (39).

From this, some considerations emerge that should be considered in order to reduce chronic inequalities in the long term:

- Strengthen vocational training and facilitate access to higher education by generating new measures to reconcile work, studies, and family.
- Generate a strong productive fabric to avoid low-skilled and precarious jobs.
- Promote community work to strengthen networks, relationships, and community ties.
- Promote and improve the prevention and promotion of emotional wellbeing.
- Increase and improve care for people with emotional distress and psychosocial risk factors to avoid or minimize the appearance of mental and physical health problems.

It is necessary to break with the economistic view of social exclusion and to axiomatize the relational and salutogenic dimension of social exclusion and well-being in policies and interventions to reduce social inequalities in health (40).

On a practical level, the results indicate the need to enhance the dimensions that have proved to be revealing in the proposed model and have the potential to become good resilient resources for social intervention specialists. Based on these results, the proposal is to design psychoeducational interventions and projects based on health promotion, cognitive restructuring, and acceptance techniques, strengthening of social support and social relationships, which includes and encourages the role of community action and the promotion of emotional well-being.

All these results are consistent with the Social Determinants of Health approach in recognizing the role of social inequalities in health and in finding a close relationship between health status and people’s opportunities and resources according to their class, gender, territory or ethnicity (8) and the SEKN model, that best explains the relationship between social inclusion - social exclusion and social inequality in health. This model focuses on the unequal power relations established in the four social dimensions (political, social, cultural and economic) and highlights the dynamics that can generate processes of differential exposure in mental health conditions.

Similarly, the conceptual model of the production of health inequalities by (41) and the socio-psychological model by Barrón and Sánchez (16) show how social position is influenced by social and environmental factors and these, in turn, are related to psychological and psychosocial mediating factors that influence mental health states and well-being.

It is assumed that the sample size (M1 *n* = 107; M2 *n* = 103) is the main limitation of the study. However, the sample size is larger than other studies published with similar characteristics. Studies that analyze the population in social exclusion in general are non-existent, and those with a large sample are very rare. Mostly, studies are based on specific groups and profiles and are usually focused on specific issues.

Moreover, it could be very interesting to propose a longitudinal design or use the method of time series to compare moments and generational cohorts, verify the influence of different environmental contexts, and study evolutionary changes.

Although predictive models do not establish a causal link between psychological and social well-being variables and coping styles, it has been found that there are factors of psychological and social well-being and coping styles, with significant levels of variance, that should be considered as stress-buffering factors in trajectories of social inclusion and exclusion.

In conclusion, the results are intended to guide anticipatory policies and implement reactive policies or strategies with innovative bases to contribute to achieving the Sustainable Development Goals (SDGs), more specifically; in advocacy to address poverty and social exclusion, work to reduce inequality (SDG10), impact on improving health and well-being, ensure quality education (SDG4), promote gender equality (SDG5) and decent work (SDG8).

## Data availability statement

The raw data supporting the conclusions of this article will be made available by the authors, without undue reservation.

## Ethics statement

Ethical review and approval was not required for the study on human participants in accordance with the local legislation and institutional requirements. Written informed consent to participate in this study was provided by the participants’ legal guardian/next of kin.

## Author contributions

All authors listed have made a substantial, direct, and intellectual contribution to the work and approved it for publication.

## Conflict of interest

The authors declare that the research was conducted in the absence of any commercial or financial relationships that could be construed as a potential conflict of interest.

## Publisher’s note

All claims expressed in this article are solely those of the authors and do not necessarily represent those of their affiliated organizations, or those of the publisher, the editors and the reviewers. Any product that may be evaluated in this article, or claim that may be made by its manufacturer, is not guaranteed or endorsed by the publisher.
